# Supplementary material for: A phenotypic Caenorhabditis elegans screen identifies a selective suppressor of antipsychotic-induced hyperphagia
Source: Nat Commun. 2018 Dec 10;9:5272. doi: 10.1038/s41467-018-07684-y (PMC6288085; doi:10.1038/s41467-018-07684-y)
Supplement: Supplementary file 1 — Supplementary Information [file 41467_2018_7684_MOESM1_ESM.pdf]

A Phenotypic Caenorhabditis elegans Screen Identifies a Selective Suppressor of Antipsychotic-induced Hyperphagia

Anabel Perez-Gomez[1,2‡], Maria Carretero[1,2‡,] Natalie Weber[3], Veronika Peterka[3], Alan To[1,2], Viktoriya Titova[1, 2], Gregory Solis[1, 2], Olivia Osborn[3*] Michael Petrascheck[1,2*]

‡ These authors contributed equally to this work
* These authors jointly supervised this work

Correspondence should be addressed to M.P (pscheck@scripps.edu) or O.O (oosborn@ucsd.edu)

Supplementary figure 1.

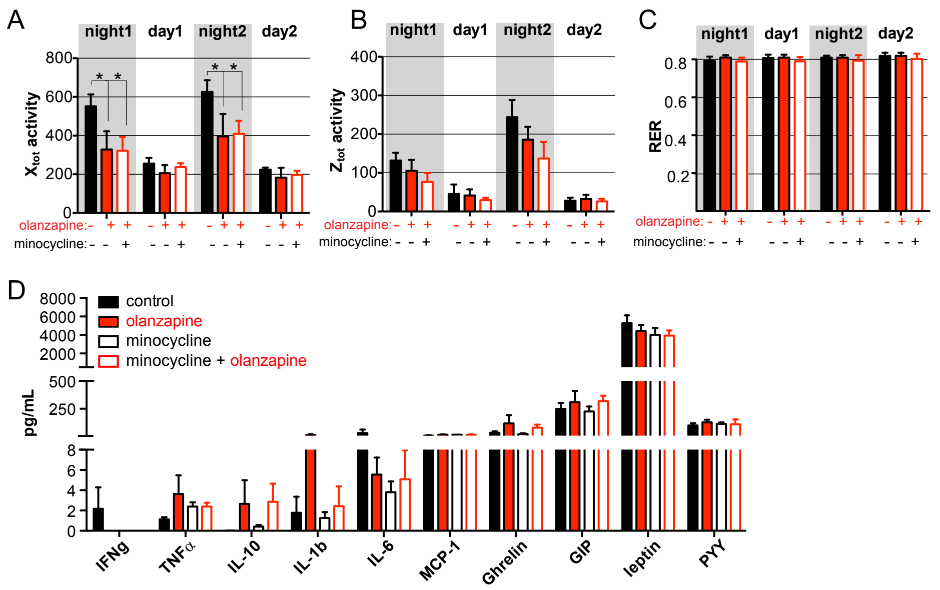

Supplementary Figure 1. Metabolic chamber analysis and comparison of levels of inflammatory cytokines and gut-derived hormones in chronic mouse study. A-C. Metabolic chamber analysis of (A) total activity at x-axis activity, (B) total activity at z-axis (ZTOT) and (C) respiratory exchange ratio (RER). Data are presented as average per group +/- S.EM. in either dark cycle or light cycle, n=4 per group. D. Comparison of levels of inflammatory cytokines and gut-derived hormones in chronic mouse study after olanzapine (OLZ), minocycline (MINO), control (CTRL) or olanzapine + minocycline (OLZ+MINO) treatment (n= 8 per group,*p<0.05 indicates significance using Two-way ANOVA with Tukey multiple comparison test). Source data are provided as a Source Data file

Supplementary figure 2.

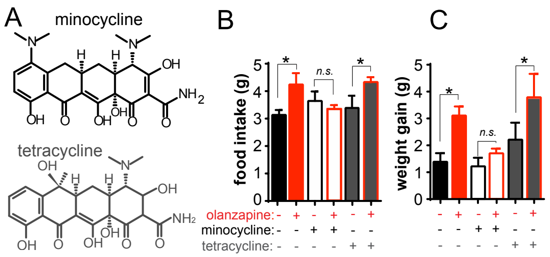

Supplementary Figure 2. Comparison of the effect of co-treatment with tetracycline or minocycline on olanzapine-induced food intake and weight gain. Co-treatment of tetracycline with olanzapine did not blunt olanzapine-induced food intake or weight gain. (A) Structure of minocycline and tetracycline. (B) Food intake and (C) weight gain in female mice treated for 2 weeks with control (CTRL) diet alone, olanzapine (OLZ), minocycline (MINO), or olanzapine + minocycline (OLZ+MINO), tetracycline (TETRA) or tetracycline + olanzapine (TOLZ). N= 6-8 per group, *p<0.05, One-way ANOVA with uncorrected Fishers LSD test. Source data are provided as a Source Data file.

Supplementary figure 3.

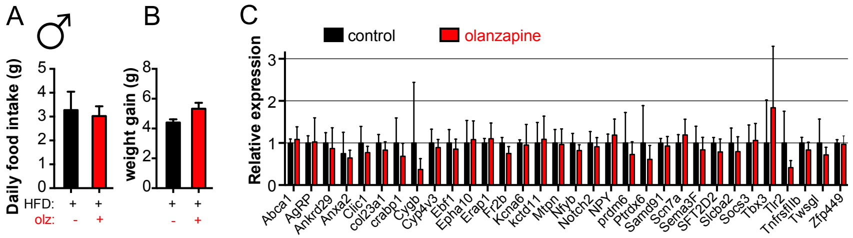

Supplementary Figure 3. Olanzapine treatment in male mice. A-B.Male mice are resistant to olanzapine induced hyperphagia (A) and weight gain (B). C. Gene expression changes in male hypothalamus (n=7 per group). Data are presented as average +/-S.E.M. Source data are provided as a Source Data file.
