# Supplementary material for: A phenotypic Caenorhabditis elegans screen identifies a selective suppressor of antipsychotic-induced hyperphagia
Source: Nat Commun. 2018 Dec 10;9:5272. doi: 10.1038/s41467-018-07684-y (PMC6288085; doi:10.1038/s41467-018-07684-y)
Supplement: Supplementary file 3 — Description of Additional Supplementary Files [file 41467_2018_7684_MOESM3_ESM.pdf]

Description of Additional Supplementary Files

File Name: Supplementary Data 1
Description: Number of animals, lysis factors, food intake and names of drugs tested in the screen in Fig.2B.

File Name: Supplementary Data 2
Description: Number of animals tested for Fig. 1, 2, 4.
